# Validation of a bladder symptom screening tool in women with incontinence due to overactive bladder

**DOI:** 10.1007/s00192-014-2417-7

**Published:** 2014-05-24

**Authors:** Linda Cardozo, David Staskin, Brooke Currie, Ingela Wiklund, Denise Globe, Manuel Signori, Roger Dmochowski, Scott MacDiarmid, Victor W. Nitti, Karen Noblett

**Affiliations:** 1 NHS, Department of Urogynaecology, King’s College Hospital, Denmark Hill, London, SE5 9RS UK; 2 Tufts University School of Medicine, Boston, MA USA; 3 Outcomes Research, Evidera, Bethesda, MD USA; 4 Global Health Outcomes Strategy and Research, Allergan, Inc., Irvine, CA USA; 5 Global Strategic Marketing, Allergan, Inc., Irvine, CA USA; 6 Vanderbilt University, Nashville, TN USA; 7 Alliance Urology, Greensboro, SC USA; 8 New York University School of Medicine, New York, NY USA; 9 UC Irvine Medical Center, Irvine, CA USA

**Keywords:** Overactive bladder, Reliability, Sensitivity, Specificity, Urgency urinary incontinence, Validity

## Abstract

**Introduction and hypothesis:**

The Actionable Bladder Symptom Screening Tool (ABSST) was initially developed to identify patients with multiple sclerosis (MS) who could benefit from lower urinary tract assessment and treatment. Assessment of the measurement properties of the ABSST, including its ability to identify patients experiencing bladder symptoms related to overactive bladder (OAB), was undertaken in a general female population.

**Methods:**

One hundred women completed the ABSST, OAB Questionnaire Short Form (OAB-q SF), and a patient global impression of severity (PGI-S) scale. Half of the sample had urgency urinary incontinence (UUI), while the other half did not. Descriptive statistics, reliability, and validity were examined, as was sensitivity and specificity of the previous cut-off score established in MS.

**Results:**

Fifty-three women with UUI/OAB and 47 controls took part (71.0 % Caucasian). Patients with UUI/OAB were older (54.6 vs 40.4 years), had a higher body mass index (31.1 vs 26.4 kg/m^2^), and more comorbid conditions. The Cronbach’s alpha reliability of ABSST was 0.90. High correlations with OAB-q SF Symptom Bother and Health Related Quality of Life (*r* = 0.83 and −0.81 respectively) supported concurrent validity. Using the PGI-S severity scores as a reference, the ABSST was able to distinguish patients with differing severity levels (known-group validity). Physician assessment of the need for further evaluation/treatment showed sensitivity (79 %) and specificity (98 %), supporting a cut-off score of ≥3.

**Conclusions:**

The previous MS ABSST scoring algorithm was validated in a non-neurogenic female population. ABSST is a reliable, valid, and sensitive tool for screening women with UUI/OAB.

## Introduction

The Actionable Bladder Symptoms Screening Tool (ABSST) was developed following the current regulatory standards [1] as a screening instrument to identify patients who could benefit from lower urinary tract assessment and/or possibly treatment. A scoring algorithm and cut-off score for recommending further clinical evaluation and treatment was originally validated in a multiple sclerosis (MS) population with symptomatic neurogenic detrusor overactive (NDO) bladder [2]. Although the ABSST was found to be effective in screening patients with MS, it has not been used or validated in other clinical populations suffering from urinary problems.

Overactive bladder (OAB) is defined as “urinary urgency, usually with frequency and nocturia, with or without urgency urinary incontinence (UUI), in the absence of urinary tract infection (UTI) or other obvious pathology” [3]. The condition is highly prevalent and is associated with significant economic burden and lower health-related quality of life (HRQoL) [4–7]. Among adult women aged ≥40 in the United States (USA), OAB is estimated to affect between 33 % and 43 % of women [8]. The total cost of OAB in the USA was estimated to be $65.9 billion in 2007 [5], 22.1 % of which was accounted for by indirect costs. In a recent study on the impact of urinary incontinence in patients with OAB, Tang et al. concluded that urinary incontinence was associated with clinically and statistically poorer general and disease-specific HRQoL, impaired work productivity and activity, and statistically higher rates of OAB-related surgery, hospitalizations, physician visits, and pad use [9].

Despite the negative impact of OAB on HRQoL, a recent registry-based, online survey study conducted across multiple countries showed that a substantial proportion of patients never consulted a physician regarding their bladder symptoms. Moreover, the study found that those patients who did consult a physician waited a number of years before doing so and generally had to initiate the consultation themselves [10]. Another survey conducted among a Nordic population suffering from lower urinary tract dysfunction found that only 8 % speak freely about their condition, while 36 % do not talk to anyone about it [11].

Given the high prevalence of OAB in women [8], in conjunction with the fact that many patients fail to mention their problems during clinical consultations, women may benefit from screening for symptoms related to OAB, including UUI. If distributed to gynecology offices for completion by patients prior to consultation, the ABSST could help to facilitate communication between patients and healthcare providers, which may not happen otherwise. Prior to the development of the ABSST, no publically available instrument used for screening a general female population for lower urinary tract problems had been developed relating to current best practices and regulatory standards [1, 12, 13]. Thus, development of the ABSST was meant to fill this critical gap.

Prior to using the ABSST to screen a more general female population, it is important to evaluate its ability to identify patients experiencing lower urinary tract symptoms (LUTS). Thus, this study evaluated the reliability and validity of the ABSST, as well as the sensitivity and specificity of the cut-off score previously established in MS for recommending further urogynecological assessment and/or treatment. Specifically, this study aimed to evaluate the ability of the ABSST to identify individuals experiencing LUTS related to OAB in a general female population presenting in gynecology offices.

A key secondary objective of the study was to assess the content validity of the ABSST in the general female population through cognitive interviewing techniques. The development of the ABSST was based on a foundation of extensive qualitative work, including a review of the literature, concept elicitation interviews with MS patients, expert clinician input, and cognitive interviews with MS patients [2]. Given the extensive qualitative work previously conducted, it was decided that concept elicitation interviews would not be necessary in the new, general female population; however, confirming the screener’s content validity through cognitive interviewing was considered an important step.

## Materials and methods

This was a prospective, observational study that involved a single study visit with 100 female patients recruited from six gynecology clinics located across the USA. Clinic staff identified potential study participants through database and chart reviews and then, using a standard recruitment and screening script, contacted prospective participants to gauge interest in participation and to ascertain eligibility for the study. Enrolled patients were women aged ≥18 years who had no history of diabetes. All study participants were required to read, speak, and write English. Approximately half of the sample had either a patient report or chart confirmation of UUI (International Classification of Diseases, Ninth Revision, Clinical Modification [ICD-9-CM] code 788.63) due to OAB (ICD-9-CM code 596.5). The remaining sample represented the control arm, which had no documented history or patient report of UUI or OAB. If patients had a history of any of the following, they were excluded from study participation: urethral stricture; genitourinary tuberculosis; bladder calculi; pelvic radiation; pelvic surgery within the past 6 months; bladder cancer; pelvic organ (hymen or below) prolapse; interstitial cystitis; neurological diagnosis (e.g., MS, Parkinson’s); current UTI; indwelling catheter. Recruitment to the study was conducted on a continuous basis until 100 eligible patients were enrolled and had completed the study. Institutional review board (IRB) approval for the study at five of the study centers was obtained through Shulman IRB on 25 May 2012; IRB approval for the sixth site was obtained locally through Virtua Health General IRB. The study’s data collection period began on 11 June 2012 and ended on 10 August 2012.

During their study visit, patients completed the ABSST, the OAB Questionnaire Short Form (OAB-q SF), a patient global impression of severity (PGI-S) scale, and a sociodemographic questionnaire. The recruiting site investigators completed a urogynecology assessment form (UAF) and a clinical form following each patient’s study visits. The information collected on the sociodemographic and clinical forms was used to describe the population, whilst the results derived from the OAB-q SF, the PGI-S, and UAF were used for validation purposes in addition to characterization of the population. The study measures are described in detail below.

### Actionable Bladder Symptoms Screening Tool

The Actionable Bladder Symptoms Screening Tool (ABSST) includes eight items that ask about micturition frequency, leakage, urgency, and nighttime voiding and the impact on social relations, work interference, and embarrassment. The ABSST utilizes a four-point Likert scale and a 7-day recall period. The ABSST includes a final question, which asks if the respondent would like to receive help for their bladder problems (yes/no). As previously established in an MS population, the ABSST score is calculated as the number of positive responses in the blue shaded area of the form (see Appendix), where a score of ≥3 (range 0–8) indicates the need for further urogynecological evaluation and/or treatment [2].

### Overactive Bladder Questionnaire Short Form

The Overactive Bladder Questionnaire Short Form (OAB-q SF) comprises a six-item Symptom Bother scale and a 13-item HRQoL. The HRQoL scale is divided into the three following subscales: coping (five items), sleep (three items), and emotional social (five items). The recall period is over the previous 4 weeks, and it uses a six-point graded Likert-type scale. The OAB-q SF has been well documented for reliability and validity across domains [14].

### Patient Global Impression of Severity

The Patient Global Impression of Severity (PGI-S) is a global index that may be used to rate the severity of a specific condition (a single-state scale) and was validated in women with stress urinary incontinence [15]. The PGI-S was adapted for this study and is a single question asking the patient to rate the severity of her UUI and/or OAB symptoms on a scale of 1 (no UUI or OAB) to 5 (very severe).

### Sociodemographic questionnaire

Study participants completed a brief questionnaire that collected information about basic sociodemographic characteristics, including month and year of birth, gender, ethnicity, living situation, highest level of education attained, and employment status. In addition to the sociodemographic questions, the form included several clinical questions to capture information on the patient’s comorbid conditions (e.g., depression, hypertension), medications used to treat any OAB symptoms, and general history of any urinary problems.

### Clinical form

Study staff from the recruiting clinical site completed a clinical form (developed specifically for the study) to capture additional clinical information for each study participant, including height, weight, and reason(s) for seeking medical consultation/treatment at that particular clinical site.

### Urogynecology Assessment Form

An assessment form was completed by the recruiting clinical site’s principle investigator (treating physician) to document the investigator’s opinion on whether or not he/she would refer the patient to a urologist or urogynecologist based on the patient’s responses to the ABSST, and which item(s) on the patient-completed ABSST led to the investigator’s referral decision. The Urogynecology Assessment Form (UAF) was developed specifically for this study.

In addition to completing these forms, a subset of 10 patients with OAB and UUI also participated in a one-to-one cognitive interview during their study visit. The purpose of the interview was to assess the content validity of the ABSST by understanding how patients described their symptom experience associated with UUI/OAB and assessing patient comprehension and understanding of the ABSST instructions, items, response options, and recall period.

### Analysis

Quantitative analyses began with summarizing the demographic and clinical characteristics of the study participants for the total sample and by UUI/OAB status, followed by descriptive statistics of the patient-reported outcome (PRO) measures used in the study. To confirm the psychometric properties of ABSST items, their performance was assessed through examination of descriptive statistics (mean, standard deviation, floor and ceiling effect, and percentage of missing response), inter-item correlation, and internal consistency reliability. Concurrent validity was assessed by the correlation of ABSST with OAB-q SF and with patient-reported experience of bladder or urinary problems. Known-group validity was assessed by stratifying participants into disease severity groups, as determined by the patient-completed PGI-S and examining whether these groups had significantly different scores on the ABSST by analysis of variance. Known-group validity was also assessed by an independent group *t* test on mean ABSST total scores between patients with and those without UUI/OAB. The sensitivity and specificity (including positive and negative predictive values) of the ABSST cut-off score was assessed using clinician urogynecological assessment as the criterion. Finally, a logistic regression analysis was conducted and the results were used to plot the receiver operating characteristic (ROC) curve.

A content analysis approach was taken to analyze data from the cognitive interviews, using ATLAS.ti qualitative data analysis software (ATLAS.ti; version 7.0). A coding dictionary was developed to capture emergent comments made by patients regarding experiences with UUI/OAB symptoms, as well as comments regarding the clarity, content, relevance, and consistency in the interpretation of ABSST instructions, items, response options, and recall period. Interview transcripts were coded to highlight patient responses regarding the comprehension, relevance to their experience with symptoms, and ease or difficulty of selecting a response.

Saturation of concepts was evaluated by reviewing the emergent part of the interviews and documenting for each patient whether these concepts emerged spontaneously or were considered relevant to experiences with UUI/OAB after the interviewer probed about them. It was understood from the outset of the study that the small cognitive interview sample size may not prove sufficient for determining true saturation of all concepts.

## Results

One hundred women were enrolled and completed the study. Approximately half of the sample (*n* = 53; 53.0 %) had UUI/OAB, while the other half (*n* = 47; 47.0 %) did not. The majority of the women were Caucasian (71.0 %) and were employed either full time (56.0 %) or part time (21.0 %). The sample was relatively highly educated with 34.0 % of participants indicating that they had a postgraduate degree, 11.0 % had a college degree, and 30.0 % reported having completed some college education. Comparing the UUI/OAB arm with the controls, the UUI/OAB sample was older, with a mean age of 54.6 ± 11.6 years compared with 40.4 ± 14.3 years for the non-UUI/OAB group. No other significant sociodemographic differences were found between the two groups (Table 1).

**Table 1:** Patient demographic and clinical characteristics

Characteristic	UUI/OAB (*n* = 53)	Non-UUI/OAB (*n* = 47)	Total (*N* = 100)
Mean age (±SD)	54.6 (±11.6)	40.4 (±14.3)	47.9 (±14.7)
Gender, *n* (%)			
Female	53 (100.0)	47 (100.0)	100 (100.0)
Racial background, *n* (%)			
White	38 (71.7)	33 (70.2)	71 (71.0)
Black or African–American	9 (17.0)	10 (21.3)	19 (19.0)
Employment status, *n* (%)			
Full-time work	30 (56.6)	26 (55.3)	56 (56.0)
Part-time work	10 (18.9)	11 (23.4)	21 (21.0)
Retired	8 (15.1)	4 (8.5)	12 (12.0)
Education level, *n* (%)			
Secondary/high school	9 (17.0)	10 (21.3)	19 (19.0)
Some college education	19 (35.8)	11 (23.4)	30 (30.0)
College degree	5 (9.4)	6 (12.8)	11 (11.0)
Postgraduate education	16 (30.2)	18 (38.3)	34 (34.0)
Comorbid conditions, *n* (%)			
High blood pressure (hypertension)	19 (35.8)	8 (17.0)	27 (27.0)
High cholesterol	15 (28.3)	3 (6.4)	18 (18.0)
Anxiety	10 (18.9)	6 (12.8)	16 (16.0)
Depression	12 (22.6)	2 (4.3)	14 (14.0)
Current bladder medication, *n* (%)			
None	41 (77.4)	47 (100.0)	88 (88.0)
Solifenacin succinate (VESIcare®)	4 (7.5)	0	4 (4.0)
Mean BMI, kg/m^2^ (±SD)	31.1 (±6.6)	26.4 (±5.0)	28.9 (±6.3)

*BMI* body mass index, *OAB* overactive bladder, *UUI* urgency urinary incontinence

The mean duration of self-reported urinary or bladder problems for the UUI/OAB group was 6.8 years, with the majority (*n* = 41; 77.4 %) reporting that they did not take any medication for their bladder problems. Comparing the UUI/OAB group with controls, UUI/OAB patients had a higher body mass index (31.1 vs 26.4) and reported higher numbers of comorbidities than those without UUI/OAB, including high blood pressure (hypertension; 35.8 % vs 17.0 %), high cholesterol (28.3 % vs 6.4 %), anxiety (18.9 % vs 12.8 %), and depression (22.6 % vs 4.3 %; Table 1).

Mean scores reported by patients on the PGI-S, ABSST, and the subscales of the OAB-q SF are presented in Table 2. Mean symptom severity scores as reported on the PGI-S differed significantly between groups, with the UUI/OAB group having a higher overall mean score (3.0 vs 1.2). Mean scores on the OAB-q SF subscales also differed significantly between groups. Higher scores on the Symptom Bother subscale indicate greater symptom severity/bother or impact, while higher scores on the HRQoL subscale indicate better HRQoL or less impact. The mean Symptom Bother score was 46.1 ± 24.3 for the UUI/OAB group compared with 11.2 ± 16.3 for the control group. The mean HRQoL score was 66.2 ± 21.8 for the UUI/OAB sample compared with 95.6 ± 10.9 for those without UUI/OAB.

**Table 2:** Descriptive statistics for patient-completed measures

Sample/measure	Characteristic		
	Mean	±SD	Range
UUI/OAB (*n* = 53)			
PGI-S^a^	3.0	±1.0	1–5
OAB-q SF: symptom bother^b^	46.1	±24.3	0.0–100.0
OAB-q SF: HRQoL^b^	66.2	±21.8	9.2–100.0
ABSST^c^	3.3	±2.2	0.0–8.0
Non-UUI/OAB (*n* = 47)			
PGI-S^a^	1.2	±0.6	1–4
OAB-q SF: symptom bother^b^	11.2	±16.3	0.0–70.0
OAB-q SF: HRQoL^b^	95.6	±10.9	43.1–100.0
ABSST^c^	0.6	±1.0	0.0–4.0

*ABSST* Actionable Bladder Symptoms Screening Tool, *HRQoL* health-related quality of life, *OAB-q SF* Overactive Bladder Questionnaire Short Form, *PGI-S* Patient Global Impression of Severity, *UUI* urinary urgency incontinence

^a^Scores have a possible range of 1–5. Higher scores indicate greater symptom severity

^b^Scores have a possible range of 0–100. Higher scores on the symptom bother scale indicate greater symptom severity/bother or impact, while higher scores on the HRQoL subscale indicate better HRQoL or less impact

^c^Scores have a possible range of 0–8. Higher scores indicate greater symptom severity or impact

Analysis of the individual ABSST items showed no significant floor effects for the UUI/OAB patients, suggesting that the items were perceived by this group as representing relevant and important problems associated with UUI/OAB. Specifically, the floor effect across all items for the UUI/OAB group was 9.4 %, while the floor effect for the control group was 63.8 %. Similarly, in the item-to-item correlation analysis, items were in general moderately to highly correlated, ranging from 0.28 to 0.81, suggesting that all items meaningfully contributed to the score with no apparent item being redundant. The highest correlation (*r* = 0.81) was noted for the item pair: item 6 (“activities with friends and family were limited” and item 7 (“embarrassed because of bladder symptoms”). The lowest correlation (*r* = 0.28) was noted for the item pair: item 5 (“how many times do you urinate in a typical day”) and item 8 (“limited ability to work outside the home”). All item-to-item correlations were statistically significant at *p* < 0.05 (data not shown).

Internal consistency reliability for the ABSST was assessed using Cronbach’s alpha. The internal consistency reliability for the ABSST total score was excellent at 0.90 and ranged from 0.88 to 0.91 when individual items were removed with no significant increase or decrease, indicating that each item contributes to measurement of a conceptually distinct domain (data not shown).

Concurrent validity was assessed based on correlations between the OAB-q SF Symptom Bother and HRQoL subscales and participants’ self-reported bladder or urinary problems. Correlations were high for the OAB-q SF Symptom Bother and HRQoL subscales (0.83 and −0.88, respectively; *p* < 0.001) and moderate for self-reported history of urinary problems (0.63; *p* < 0.001; data not shown).

Known-groups validity was evaluated by comparing the ABSST total score by participants’ self-reported UUI/OAB severity as measured by the PGI-S, clinician diagnosis of UUI/OAB at enrollment, and patients’ self-report of experience with bladder or urinary problems (Table 3). The pair-wise comparisons for PGI-S rating were all statistically significant (*p* < 0.0001) for each severity rating showing that ABSST scores could distinguish UUI/OAB patients at various severity levels. Similarly, the independent groups *t* test comparisons between participants with UUI/OAB and those without UUI/OAB at enrollment and patients’ self-report of experience with bladder or urinary problems were both statistically significant (*p* < 0.0001).

**Table 3:** Known groups validity of ABSST

Variable/sample	*N*, mean ABSST score (±SD)	ANOVA^a^ (*p* value)	*t* test (*p* value)
PGI-S			
No urinary problems	44, 0.4 (±0.6)	87.05 (<0.0001)	N/A
Mild	18, 1.4 (±1.0)		
Moderate	20, 3.5 (±1.6)		
Severe/very severe	18, 5.1 (±1.8)		
Clinician diagnosis at enrollment			
UUI/OAB	53, 3.3 (±2.2)	N/A	7.72 (<0.0001)
Non-UUI/OAB	47, 0.6 (±1.0)		
Patient-report of bladder/urinary problems			
“Yes”	59, 3.2 (±2.2)	N/A	8.07 (<0.0001)
“No”	41, 0.4 (±0.6)		

*ABSST* Actionable Bladder Symptoms Screening Tool, *ANOVA* analysis of variance, *N/A* not applicable, *OAB* overactive bladder, *PGI-S* Patient Global Impression of Severity, *UUI* urinary urgency incontinence

^a^ANOVA with Scheffe’s post hoc comparisons: mild vs no urinary problems (*p* < 0.05), moderate vs no urinary problems (*p* < 0.0001), severe/very severe vs no urinary problems (*p* < 0.0001), moderate vs mild urinary problems (*p* < 0.0001), severe/very severe vs mild urinary problems (*p* < 0.0001), and severe/very severe vs moderate urinary problems (*p* < 0.001)

The results of the sensitivity and specificity analysis of the ABSST cut-off score of ≥3 (previously established in patients with MS) are displayed in Table 4. Among those participants who clinicians assessed as needing treatment for UUI/OAB (*n* = 43), 34 had an ABSST total score of ≥3, while 9 had a score <3; thus, sensitivity was 79.1 %. Among the group of patients who clinicians assessed as not needing treatment (*n* = 57), only 1 participant had an ABSST total score ≥3, while 56 had a score <3; thus, specificity was 98.2 %. In other words, the cut-off score of ≥3 matched the clinician’s assessment of whether or not a patient should be treated for OAB/UUI. This was consistent with the sensitivity and specificity findings for the MS population where the cut-off of ≥3 was also determined by clinicians (sensitivity 82 %, specificity 95 %; data not shown).

**Table 4:** Predictive validity of the ABSST with clinician urogynecology assessment

ABSST score ≥3 (patient)	Clinician urogynecology assessment: patient to be treated for OAB/UUI	
	Yes	No
Positive	34	1
Negative	9	56
Total	43	57
Chi-squared	64.4	
Sensitivity	79.1	
Specificity	98.2	
Positive predictive value	97.1	
Negative predictive value	86.2	

*ABSST* Actionable Bladder Symptoms Screening Tool, *OAB* overactive bladder, *UUI* urinary urgency incontinence

**Fig. 1:**
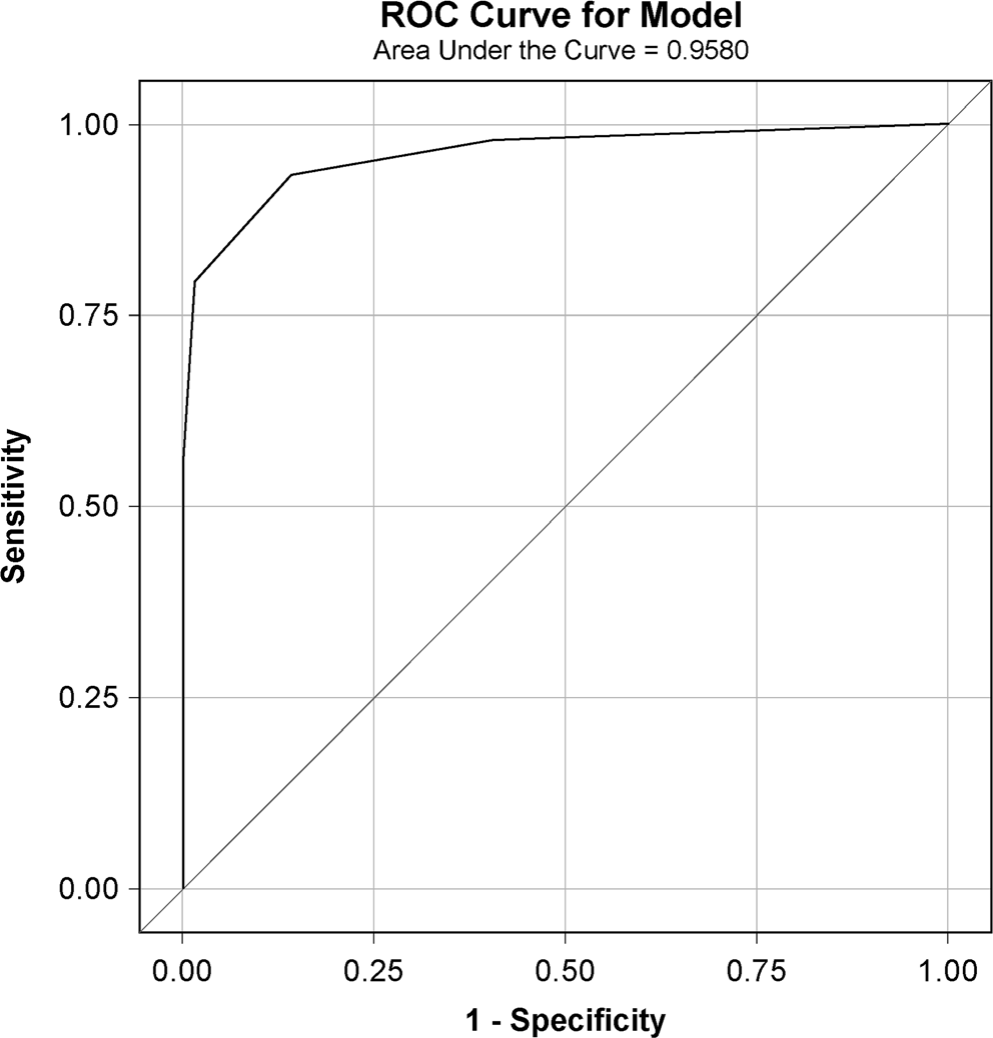
Receiver operating characteristic (ROC) curve with Actionable Bladder Symptoms Screening Tool (ABSST) total score in predicting clinician urology assessment

Figure 1 displays the ROC for the predictive validity of the ABSST using the clinician’s assessment of whether or not treatment is needed. The curvature of the curve (farther to the upper left-hand corner) and the area under the curve (also known as the c-statistics) indicate how well the ABSST total score discriminated between the positive and negative classifications.

Results from the cognitive interviews should be interpreted with some level of caution, as it is difficult to draw definitive conclusions around concept saturation with a sample size of only 10 participants. With this caveat in mind, the UUI/OAB patients generally found the ABSST items easy to understand and respond to, as well as relevant to their experiences with LUTS. No key symptoms were reported to be missing. Overall, the language used for instructions, items, and response options were considered by patients to be appropriate. The symptom impact items were found to be somewhat relevant to the female patients; however, perhaps not as pronounced as was found in previous work conducted with MS patients.

## Discussion

Based on the high prevalence and significant impact of OAB in women aged ≥40 years in the US [8], there is a need for a well-developed and validated screening tool for urinary symptoms in a general female population. Women presenting to a gynecological or general practitioner office setting with symptoms indicative of UUI/OAB are likely to suffer the same negative impacts as people with this condition in general (i.e., significantly reduced participation in social activities, increased psychological distress, and decreased quality of life). Despite a significant reduction in HRQoL in patients suffering from urinary dysfunction, many do not seek medical help, possibly out of embarrassment or because they do not believe that treatment is available, even when being seen by a clinician for other conditions [11]. It is believed that the use of a simple urinary symptom screener in a gynecological office setting may facilitate discussions between the patient and their healthcare provider, and thereby help to identify women who could benefit from treatment and facilitate discussion. Such a screener would also be useful in monitoring disease progression, as well as response to treatment.

The ABSST, a new screening tool, was first developed in patients with MS using a multifaceted and iterative approach, utilizing both qualitative and quantitative methods and ongoing input from a steering committee (including informed clinical perspective) [2]. Of note, there is no specific regulatory guidance on the development and validation of screening measures; however, the methodologies used in the development of the screening tool and in the subsequent validation work described herein closely adhered to the Food and Drug Administration’s Final PRO Guidance [1].

The primary objective of the current study was to validate the ABSST for clinicians to use to screen for LUTS in women presenting in gynecological practices. Results showed that the ABSST was a reliable instrument, as demonstrated by its high internal consistency coefficient. Moreover, the screening tool correlated highly with both the OAB-q SF Symptom Bother and HRQoL subscales, as well as with patients’ self-reported experiences of bladder or urinary problems. These correlations indicate that the ABSST assesses symptoms and impacts related to UUI/OAB. It was shown that there were significant differences in ABSST total scores between patients with different levels of self-reported UUI/OAB severity. ABSST total scores were also significantly different between patients with and those without UUI/OAB, and between patients who reported having bladder problems versus those who did not. These differences indicate that the ABSST total scores appropriately reflect the severity of UUI/OAB symptoms.

Established criteria for the evaluation of psychometric properties of a screening tool are unique to the prevalence of a condition. Concern is generally focused on specificity, as it has a greater impact on predictive values, and therefore, a reliable and valid screening tool will also have sufficient ability to discriminate patients who do not have the condition (specificity) from those who do and may need to be referred (sensitivity) [16]. Results from this study also demonstrated that a cut-off ABSST score of 3 distinguishes between patients who should be treated for UUI/OAB versus those who do not require treatment. Specifically, a total score of ≥3 was found to be both sensitive and specific to the clinician-based assessment of whether or not treatment is needed. Again, this was consistent with the sensitivity and specificity findings for the MS population, where the cut-off score of ≥3 was determined by expert clinicians. A score of 3 on the ABSST may highlight the presence of bladder symptoms consistent with OAB and facilitate critical communication between the patient and their healthcare provider and further evaluation when warranted.

Results from the one-to-one interviews conducted with a subsample in this study showed that UUI/OAB patients generally found the ABSST items easy to understand and respond to, as well as relevant to their experiences with lower urinary tract problems. Note that these qualitative results should be interpreted with some caution, as it is difficult to draw definitive conclusions around content validity with a sample of 10 patients.

Another limitation of this study may be that the women who enrolled in the study represented a group of patients who were relatively well educated and also willing to discuss their LUTS in a research setting. Thus, the results of this study may not be generalizable to groups with lower education. Additionally, study participant responses may not be reflective of patients who have not presented for treatment and/or who would be less willing to discuss their symptoms with a healthcare provider. Another study limitation to consider is that the diagnosis of OAB was based on the clinician’s report without a consistent definition across clinicians. However, since OAB is a symptom-based diagnosis, this limitation may not be significant. Finally, no inter-rater reliability of diagnosis or referral for treatment was evaluated. This is most reflective of clinical practice and appropriate given that the tool is specifically developed to screen for potential OAB and is not a diagnostic tool.

In summary, the ABSST scoring algorithm, previously established in an MS population, was validated in a general female population recruited through gynecological practices. The sensitivity and specificity results support using the same cut-off score for this general female population as in the MS population. Additionally, language used in the ABSST appears to be appropriate and the measured concepts relevant to this general female population.

The current version of the ABSST consists of items covering symptoms and impacts of bladder problems. The goal of this tool is to provide screening information useful for clinicians in identifying patients who may benefit from evaluation and treatment for OAB-related urinary symptoms. If this tool is to be adapted to assess the effect of urinary symptoms in a clinical trial or for a labeling claim, additional qualitative and potentially quantitative validation in a wider pool of patients would be recommended.

In conclusion, the ABSST is a reliable, valid, and sensitive tool that can be used to identify women who may benefit from treatment and to facilitate discussions between patients and healthcare providers regarding lower urinary tract problems.

## Appendix

Actionable Bladder Symptom Screener

Score is based on the number of positive answers in the blue-shaded portion of the questionnaire.
